# Recognition of an extralobar pulmonary sequestration during lung resection

**DOI:** 10.1186/s13019-024-02880-y

**Published:** 2024-07-13

**Authors:** Ming Zhang, Weifen Tang, Hao Shi, Xiabin Tu, Weidong Li, Zhengliang Wei

**Affiliations:** 1 Department of Cardiothoracic Surgery, Shengzhou People’s Hospital (The First Affiliated Hospital of Zhejiang University Shengzhou Branch), Shaoxing, 312400 China; 2 Operation Room, Shengzhou People’s Hospital (The First Affiliated Hospital of Zhejiang University Shengzhou Branch), Shaoxing, China; 3 Department of Cardiovascular Surgery, The First Affiliated Hospital, School of Medicine, Zhejiang University, Hangzhou, 310003 China

**Keywords:** Extralobar pulmonary sequestration, Video-assisted thoracoscopic thoracotomy, Mediastinum mass

## Abstract

**Background:**

Extralobar pulmonary sequestration is located outside the lung parenchyma and is covered by a separated pleural sac, which comprises approximately 25% of all pulmonary sequestration.

**Case presentation:**

This article reported one case of an extralobar pulmonary sequestration originated from the mesoesophagus, which was recognized and excised during a lung resection. Histologic examination revealed an ectopic lung tissue with hyperplasia of bronchioles, which was accord with an extralobar pulmonary sequestration.

**Conclusions:**

CT angiogram, ultrasound and MRI can be used to clarify the diagnosis and detect the abnormal feeding arteries of extralobar pulmonary sequestration. Carefulness should be taken while dissecting and ligating the potential feeding arteries. Endovascular occlusion might be an alternative option to surgery.

## Background

Pulmonary sequestration is defined as a non-functional pulmonary tissue with no connection with the bronchial tree and receives its blood supply from the systemic circulation. The incidence of pulmonary sequestration is estimated to be 0.15–1.8%, and it accounts for 0.15–6.4% of congenital pulmonary malformations [1]. Extralobar pulmonary sequestration is located outside the lung parenchyma and is covered by a separated pleural sac, which comprises approximately 25% of all pulmonary sequestration [2]. In this article, we reported one case of an extralobar pulmonary sequestration originated from the mesoesophagus, which was recognized and excised during a lung resection.

## Case presentation

**Fig. 1:**
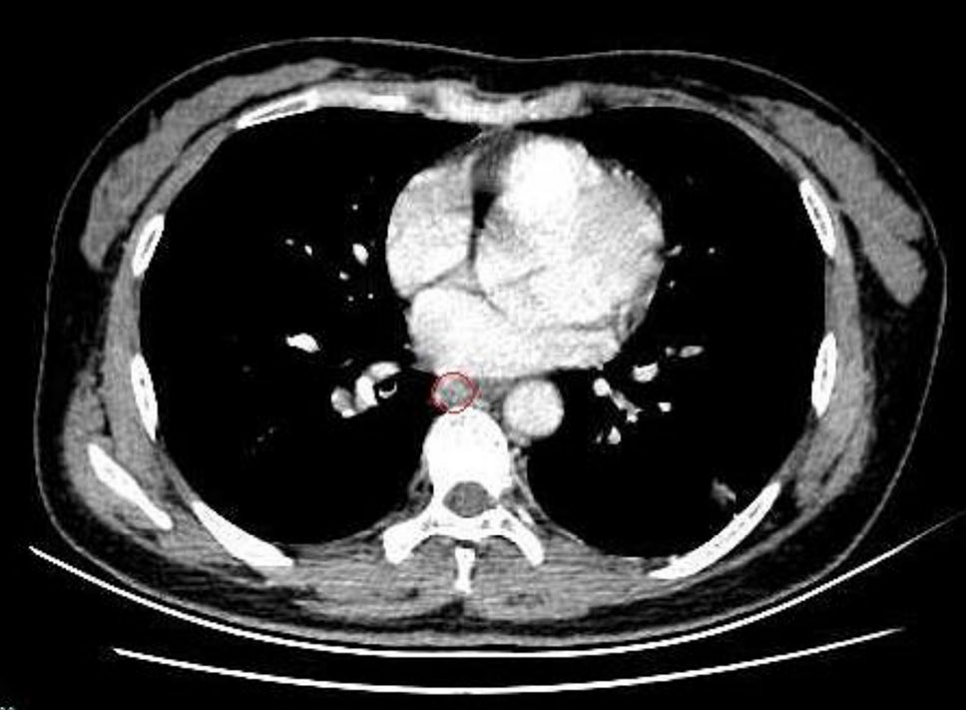
Chest CT scan revealed a sharply marginated mass, 2 × 3 cm in size, in the posterior mediastinum (marked in red circle)

**Fig. 2:**
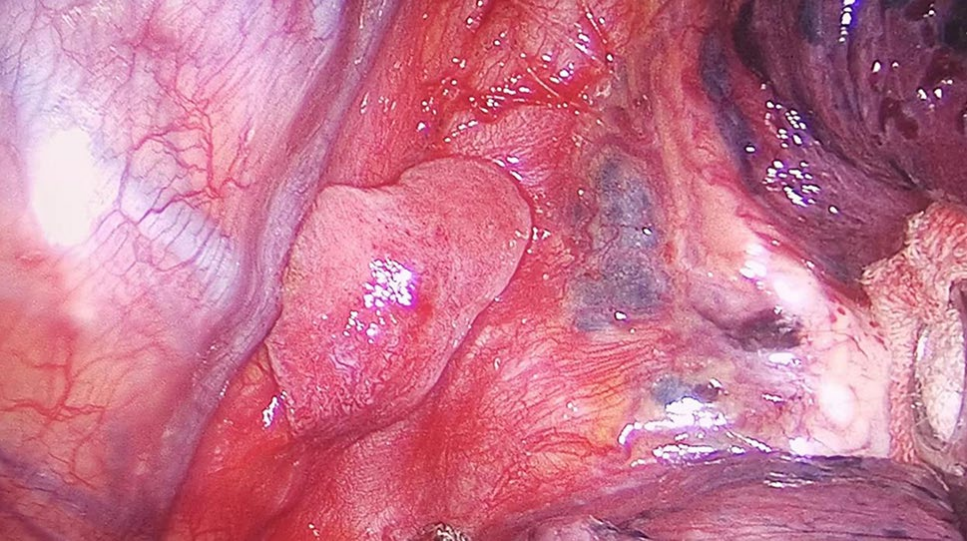
Intraoperative view of the extralobar pulmonary sequestration originated from the mesoesophagus

**Fig. 3:**
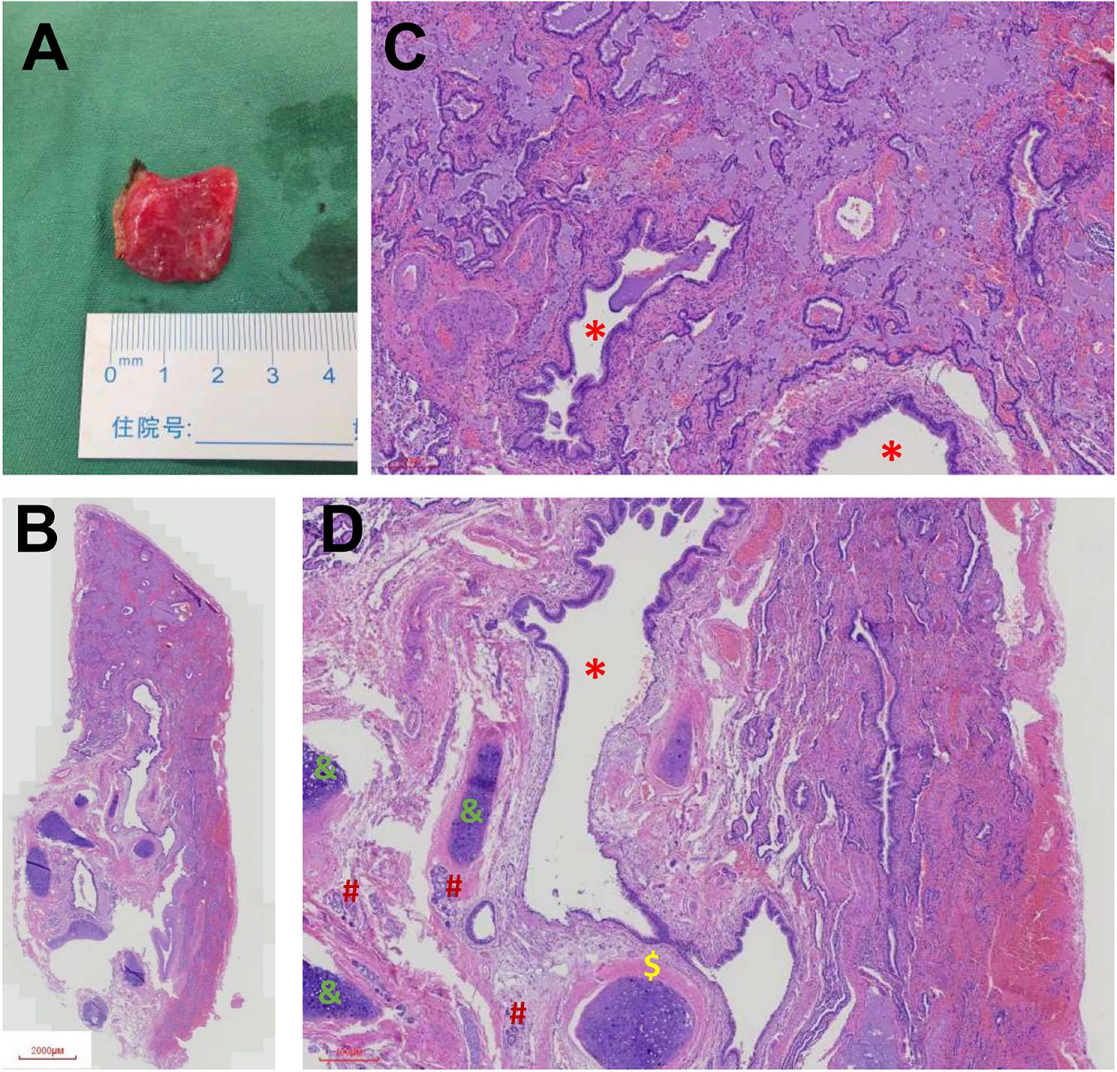
Gross morphology of the extralobar pulmonary sequestration (**A**). Histologic examination revealed an ectopic lung tissue with hyperplasia of bronchioles (*). Cartilage (&), smooth muscle ($) and submucosal glands (#) were found near the bronchus in the ectopic lung tissue (**B,C,D**)

A 52-year-old asymptomatic woman with multiple right pulmonary nodules found by CT scan 5 months ago was admitted to our hospital. Physical examination and laboratory tests showed no significant abnormalities. Chest CT scan showed multiple ground-glass opacities in the right superior and inferior pulmonary lobes, and a sharply marginated mass, 2 × 3 cm in size, in the posterior mediastinum (Fig. 1). A right-sided video-assisted thoracoscopic thoracotomy was performed. Intraoperatively, wedge resection of the right superior and inferior lung and systematic hilar and mediastinal lymph node dissection was performed. A 2.5 cm mass was found originated from the mesoesophagus at the level of 9th intercostal space. The mass was soft and pink in appearance, and had a clear boundary. The base of the mass was wide, which connected with the mesoesophagus (Fig. 2). The mass was resected completely using an endoscopic linear cutter. Histologic examination revealed an ectopic lung tissue with hyperplasia of bronchioles. Cartilage, smooth muscle and submucosal glands were found near the bronchus in the ectopic lung tissue. The diagnosis was accord with an extralobar pulmonary sequestration (Fig. 3). The patient recovered uneventfully after the operation, and was discharged on the 5th postoperative day.

## Discussion

Extralobar pulmonary sequestration usually presented between the diaphragm and the lower lobes, while only 10–15% occurred below the diaphragm [3]. In adult patients, the accompanying symptoms of extralobar pulmonary sequestration are uncommon. Torsion of the vascular pedicle could lead to acute abdominal pain [4] and hemorrhagic infarction [5]. Other symptoms include chest pain, massive hemothorax [6] and pleural effusion [7]. It has been demonstrated that more than 50% patients with extralobar pulmonary sequestration had other congenital anomalies, such as congenital diaphragmatic hernia, airway malformation and congenital heart disease [1].

In the present case, the ectopic lung tissue was found connected with the mesoesophagus, which is a rare type of extralobar pulmonary sequestration communicating with the gastrointestinal tract through a patent bronchus-like structure, known as congenital bronchopulmonary foregut malformations (CBPFMs). It has been reported that about 70% of CBPFMs were communicated with the lower esophagus [1].

The preoperative diagnosis of extralobar pulmonary sequestration is challenging, and without symptoms, it might be unrecognized for years [8]. Digital subtraction angiograpghy used to be the golden criteria to diagnose extralobar pulmonary sequestration, which can clarify the diagnosis by detecting the abnormal vessels connecting the systemic circulation and pulmonary sequestration, while in recent years, it has been substituted by noninvasive method such as computed tomographic angiograms [1]. Ultrasound can also be used in the diagnosis of pulmonary sequestration. Extralobar pulmonary sequestration may present as triangular-shaped hypo-echo or equal-echo mass under ultrasound, and color doppler can allow identifying the systemic arterial supply to the lesion [1]. It has been suggested that MRI has advantages in diagnosis of extralobar pulmonary sequestration accompanied by vascular pedicle torsion, which can display the subpleural adipose tissue clearly, and distinguish the relationship between pulmonary sequestration and normal lung tissue or pleura. Hemorrhagic pulmonary sequestration usually presented as T1W1 hyperintensity [9]. 3D-CT and MR angiography play a key role for preoperative assessment of the systemic arterial supply of pulmonary sequestration, which can reduce the risk of vascular injury and hemorrhage during dissection and ligation of feeding arteries [1].

Surgical resection is the treatment of choice for extralobar pulmonary sequestration. The safe resection of an extralobar pulmonary sequestration requires carefully dissection of the potential feeding arteries, while not paying enough attention in this regard might lead to severe bleeding. The surgical mortality has been reported in 1% of cases secondary to intraoperative hemorrhage from an unrecognized arterial supply [10]. The abnormal feeding arteries of pulmonary sequestration are friable, and usually combined with atherosclerosis, which may lead to massive hemorrhage when dissecting, traction or ligating these vessels. Controlled hypotension can be induced when ligating feeding arteries to reduce the risk of massive hemorrhage. Endovascular occlusion of the abnormal feeding arteries can reduce blood supply to the sequestered lung tissue, leading to necrosis, fibrosis and involution of the sequestration, which can be a useful approach to minimize the risk of intraoperative hemorrhage, as well as an alternative to surgery [11].

## Conclusion

Extralobar pulmonary sequestration is a congenital pulmonary malformation which located outside the lung parenchyma and receives its blood supply from the systemic circulation. CT angiogram, ultrasound and MRI can be used to clarify the diagnosis and detect the abnormal feeding arteries. Surgical resection is the treatment of choice for extralobar pulmonary sequestration. Carefulness should be taken while dissecting and ligating the potential feeding arteries. Endovascular occlusion might be an alternative option to surgery.

## Data Availability

Please contact author for data requests.
